# Ecology and Population Structure of Two Sympatric Rodents in a Neotropical Forest of Southeastern Brazil

**DOI:** 10.3390/life15111642

**Published:** 2025-10-22

**Authors:** Ricardo Bovendorp, Gabriela Moreno, Matheus Feitosa, Alexandre Percequillo

**Affiliations:** 1 Laboratório de Ecologia Aplicada à Conservação, Departamento de Ciências Biológicas, Universidade Estadual de Santa Cruz, Ilhéus 45662-900, BA, Brazil; gabrielamc9719@gmail.com (G.M.); matheus.l.b.feitosa@gmail.com (M.F.); 2 Laboratório de Mamíferos, Departamento de Ciências Biológicas, Escola Superior de Agricultura “Luiz de Queiroz” (ESALQ), Universidade de São Paulo, São Paulo 05508-220, SP, Brazil; percequillo@usp.br

**Keywords:** *Euryoryzomys russatus*, *Sooretamys angouya*, population dynamic, Atlantic Forest, diet

## Abstract

Rodents are the most diverse group of mammals, yet the natural history of many species remains poorly understood due to their elusive behavior. In this study, we examined the population structure, home range, space use, and food selection of two sympatric sigmodontine rodents, *Euryoryzomys russatus* and *Sooretamys angouya*, in the Morro Grande Forest Reserve, Brazil. *E. russatus* was more abundant than *S. angouya*, with its capture rates influenced by temperature. In contrast, the population variation of *S. angouya* showed no clear relationship with the assessed biotic (fruits and arthropods) or abiotic factors (temperature and precipitation), suggesting different primary regulatory factors for its population or a more generalist ecological strategy. The two species exhibited vertical stratification in space use: *S. angouya* displayed scansorial and arboreal locomotion, while *E. russatus* remained strictly terrestrial. Home range size, space use, and mobility were primarily influenced by resource availability, reproductive cycles, and individual body size. Our findings provide insights into the life strategies of these species, specifically regarding their vertical stratification in space use and their distinct responses to environmental resource fluctuations, enhancing our understanding of how sympatric rodents navigate shared spatial and temporal environments.

## 1. Introduction

Non-volant small mammals represent one of the most ecologically diverse groups of mammals in tropical forests [1,2]. Most species are nocturnal and solitary, primarily feeding on arthropods, fruits, and seeds [3]. In Brazilian forests, small mammals comprise marsupials (Didelphimorphia: Didelphidae) and small rodents (Rodentia: Sciuridae, Cricetidae, Caviidae, Ctenomyidae, and Echimyidae), encompassing approximately 335 species. Despite their diversity, at least 46 species are classified as Data Deficient (DD) on the IUCN Red List [4] or the São Paulo Red List [5], reflecting a significant gap in knowledge regarding their natural history, population sizes, and geographic distributions—key factors in assessing extinction risks [4]. Additionally, the mechanisms facilitating their coexistence within the same forest strata remain poorly understood [6,7,8]. Among these assemblages, scansorial and arboreal species play a crucial role, and vertical stratification in space use is a key factor enabling the coexistence of species with similar body sizes and potentially overlapping diets in the Atlantic Forest [7,9,10].

Sharing ecological niches in forest habitats of the São Paulo Plateau, two sympatric and similarly sized species within the Oryzomyini tribe—*Euryoryzomys russatus* (Wagner, 1848) and *Sooretamys angouya* (Fischer, 1814)—exhibit predominantly nocturnal habits and are commonly found in both continuous and fragmented forest landscapes [11,12]. Although both species are locally abundant, they display contrasting ecological profiles in terms of habitat preference and sensitivity to fragmentation. *Euryoryzomys russatus*, with an average body mass of 70–100 g, was recently classified as Near Threatened (NT) in the state of São Paulo [5] and is typically restricted to large continuous forests or well-connected fragments, suggesting high habitat specificity. It is a typical Atlantic Forest dweller, occurring in eastern Brazil—from moist forest enclaves within the Caatinga in the northeast to the northern region of Rio Grande do Sul—including the eastern coastline and continental islands of São Paulo and Rio de Janeiro states and extending into Misiones Province in Argentina and eastern Paraguay [13]. In contrast, *S. angouya*, with an average body mass ranging from 84 to 161 g, is classified as Least Concern (LC) and shows greater tolerance to landscape fragmentation, often being more common in smaller or isolated remnants of Atlantic Forest in southeastern Brazil [12,13,14,15,16,17]. Globally, both species are listed as Least Concern (LC) [4]. This ecological divergence provides a valuable opportunity to compare demographic, behavioral, and spatial patterns under varying habitat conditions and seasonal dynamics, contributing to a broader understanding of the natural history and ecological strategies of coexisting small mammal species in forest landscapes.

Despite their recognized ecological importance, the natural history and ecology of *Sooretamys angouya* and *Euryoryzomys russatus* remain poorly understood, limiting our comprehension of their life-history strategies and environmental responses. To address this gap, the present study aimed to provide a detailed assessment of the seasonal population structure of these two sympatric species in the São Paulo Plateau, based on intensive monitoring conducted over 26 consecutive capture–mark–recapture sessions. Specifically, we investigated (1) how the populations of these species are structured across the three grids of the Morro Grande Forest Reserve (RFMG) throughout the year and (2) how abundance, density, and reproductive activity vary in response to the availability of food resources (fruits and arthropods) and seasonal fluctuations in temperature and precipitation. Based on known ecological traits, we formulated the following hypotheses: (1) because they belong to the same tribe and share similar ecological niches, the two species would exhibit comparable population structures, responding similarly to food availability and climatic variation; (2) higher capture and recapture rates would be associated with increased availability of fruits and arthropods; and (3) population activity for both species would be higher during the warmer and wetter periods of the year.

## 2. Methods

### 2.1. Study Area

**Figure 1:**
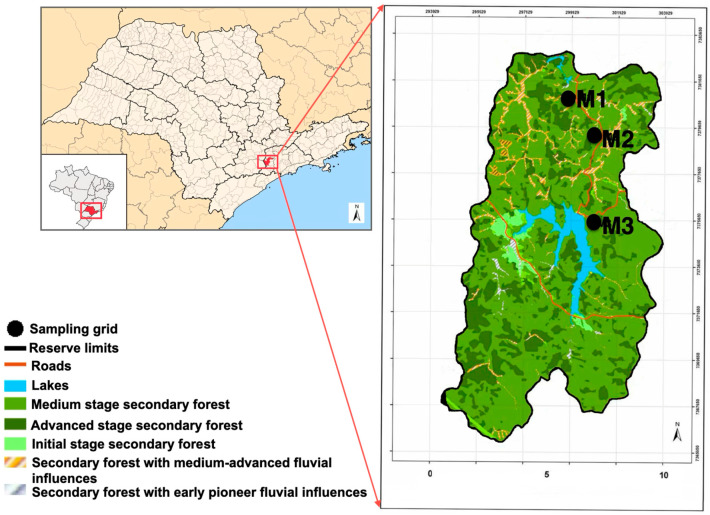
The sample grids M1, M2, and M3 chosen to capture small mammals at Reserva Florestal Morro Grande in Cotia Municipality, São Paulo, Brazil (adapted from Laboratório de Ecologia da Paisagem e Conservação—LEPaC).

The study was conducted in the Reserva Florestal Morro Grande—RFMG (23°39′–23°48′ S, 47°01′–46°55′ W), a protected area of 10.870 hectares of Dense Ombrophilous Montane Forest in Cotia, São Paulo, Brazil (Figure 1).

In this region, climate is observed; i.e., C: temperate (warm temperate/mild climate); f: no dry season (precipitation is evenly distributed throughout the year); a: hot summers (the warmest month has an average temperature above 22 °C), according to the Köppen classification system [17]. The average annual precipitation is 1339 mm, with the average of the driest month oscillating between 30 and 60 mm, while that of the wettest month varies from 150 to 200 mm [11]. Composed of a mosaic of mature and secondary forest in different stages of regeneration, the RFMG hosts 26 known species of non-volant small mammals, as well as rich assemblages of medium and large mammals [14,18].

### 2.2. Sampling Design

**Figure 2:**
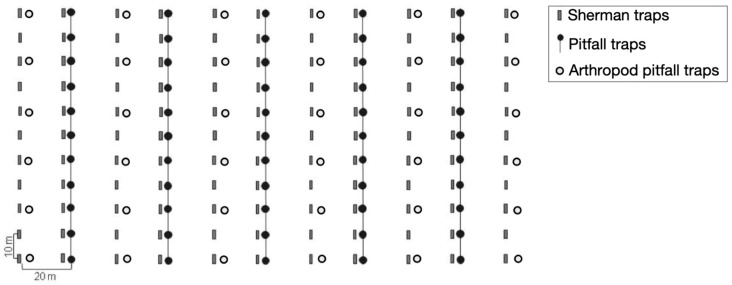
Layout of the trapping grids used for small mammal and arthropod sampling.

For this study, three 2-hectare grids (100 m × 200 m), designated as M1, M2, and M3, were established in the RFMG. These grids were placed in areas with 100% vegetation cover, each exhibiting distinct vegetation characteristics. M1 was set in a previously deforested area that has been undergoing natural regeneration without human interference since 1960, with an understory dominated by piper (Piperaceae) [19]. M2 was established on a site that experienced selective logging in the past but has also remained undisturbed since 1960 with an understory dominated by a shrub species, *Psychotria vellosiana* (Rubiaceae) [19]. M3 was located in an area subjected to selective logging over a century ago, with no human intervention since then. This site features a more structured forest, with a diverse presence of Arecaceae species and a well-developed understory with fewer shrubs [19,20]. M1, the grid closest to the edge of the RFMG, is located two kilometers from M2, while M2 is separated from M3 by four kilometers (Figure 1). These distances were chosen to analyze small mammal population dynamics and movement patterns in the area [21]. Each sampling grid consisted of 11 parallel 100-m lines, 20 m apart, with trapping stations located every 10 m. At each station, one Sherman trap (37.5 × 10.0 × 12.0 cm or 23.0 × 7.5 × 8.5 cm; H. B. Sherman Traps, Inc., Tallahassee, FL, USA) was placed on the ground. Additionally, in 5 of the 11 lines, pitfall traps (60-L cylindrical buckets, 53.0 cm in depth and 40.0 cm in diameter) connected by a 50-cm-high plastic fence were installed at each trap station. In total, each grid contained 121 Sherman traps and 55 pitfall traps. Furthermore, 36 pitfall traps for arthropod collection (400 mL plastic cups, buried at ground level) were installed per grid. These were arranged with six stations at 20 m intervals along each of the six lines that exclusively featured Sherman traps (Figure 2).

All mammal traps were baited with a mixture of sardines, peanut butter, banana, and corn meal. Traps were checked early each morning and re-baited if necessary.

### 2.3. Capture–Recapture

The spatial variation in the abundance and density of *Euryoryzomys russatus* and *Sooretamys angouya* was assessed from March 2008 to February 2010, with additional sampling sessions conducted in July 2010 and January 2011. Each grid underwent 26 capture sessions. The first session lasted four days, while subsequent sessions continued for five consecutive days until February 2010. This sampling design was specifically chosen to monitor small mammal population and community dynamics over time in the RFMG. Monthly sampling provides a temporally detailed understanding of changes in abundance, composition, and turnover in this forest ecosystem. In the extended sampling periods, data collection lasted 16 days in July 2010 and 13 days in January 2011. During the final sampling campaigns, we incorporated the spool-and-line tracking technique to study fine-scale movement and habitat use, which required extending the duration of fieldwork and adapting the standard capture-recapture protocol to accommodate the additional procedures. A small spool of thread was securely attached to the animal’s lower back using a harness made of cotton thread or surgical tape. As the animal moved through its natural habitat, the thread unspooled and left a continuous trail. This trail was later followed and mapped to record the path taken, allowing for precise measurement of movement distance, turning angles, and habitat use. The technique is minimally invasive and widely used for tracking small mammals in dense vegetation.

We had a total effort of 78.144 trap-nights. For each grid (M1, M2 and M3), the effort was 26,048 traps-night. From the total effort, 53.724 traps-night correspond to the Sherman traps, and 24.420 traps-night correspond to pitfall traps.

### 2.4. Biological Data

For all rodents captured, the following information was collected: species, tag (individual earring number—Small Animal Tags OLT; A. Hartenstein GmbH, Würzburg-Versbach, Germany), sex, sampling station, trap type, capture or recapture, body weight and length, reproductive condition, and age. We used a combination of body mass, reproductive condition, and pelage characteristics to determine age classes [22,23]. All capture, handling, and tagging protocols followed the guidelines of the American Society of Mammalogists [24].

### 2.5. Climate Data

**Figure A2:**
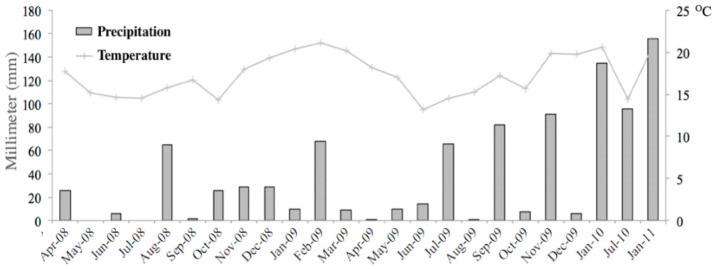
Precipitation (millimeters—mm) and temperatures (Celsius degrees—°C) recorded during the five days in the fieldwork (sampling non-volant small mammals) in Reserva Florestal Morro Grande, São Paulo, Brazil.

The climatological data were measured in each grid by a 500 mm pluviometer and by a datalogger (iButtonLink^®^, iButtonLink Technology, Whitewater, WI, USA) to record the temperature. During the 5 days of fieldwork, daily temperature and precipitation were measured over the 24 months of sampling, from April 2008 to January 2010, and 16 days in July 2010 and 13 days in January 2011, in three grids sampled. We found no difference in precipitation and temperature through the grids, and we used the average of the grids as the climatological data for each month sampled in our analysis (Appendix A, Figure A2).

### 2.6. Arthropods

The arthropods were not collected during the first 7 month (stating in September of 2008), as the sampling protocol at that time was focused exclusively on capture and re-capture on small mammals. To collect mainly terrestrial arthropods, we used small pitfall traps (see sampling design above), filled with 200 mL of 92% alcohol [25]. The pitfalls were opened during the five days of fieldwork and checked daily. The arthropods were dried in an oven at 60 °C for five days and weighed. A total of 828 arthropod samples were screened per grid. The total number of arthropod samples screened across all grids at the end of the 19 months of sampling was 2052 samples.

### 2.7. Fruits

**Figure A3:**
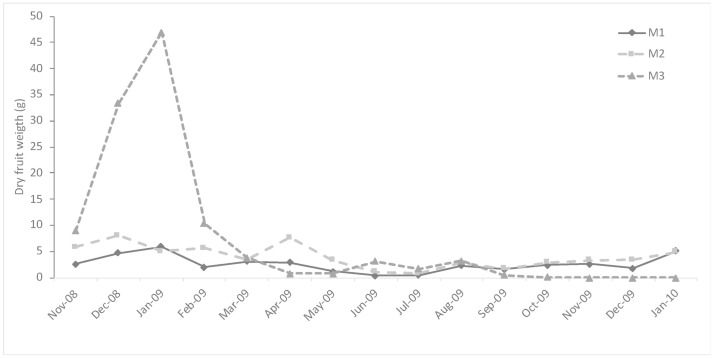
Dry fruit weight (g) sampled in each grid (M1, M2, and M3) per month.

The fruits available in the area were sampled from November 2008 to October 2009, totaling 12 months. The manual fruit sampling technique involved surveying transects (2 m wide × 2 m high) along the 100-m intercalated lines within the grids that contained only Sherman traps. These Sherman-only lines were selected due to the reduced influence on vegetation along the trapline. Only fruits in the branch trees within this defined transect area were counted, including all fruits on any tree intersecting the transect, even those outside the delimited area. Fruits on the ground were not counted due to decomposition status and/or damage, predation, and other factors that affect the fruit integrity. Subsequently, a sample of 5 to 10 fruits per tree was taken to calculate an average wet weight; this average was then multiplied by the total number of fruits visually estimated on the tree to determine the total wet fruit biomass per tree. Fruits were dried in an oven at 60 °C for seven to ten days and weighed again to calculate the total dry fruit biomass per tree. For species identification, samples of leaves, flowers, and fruits were collected from each sampled plant and identified at the Botanical Garden of the University of São Paulo, São Paulo. During the sampling period, a total of 4363 fruiting events were recorded (counting the same individual plant multiple times if it fruited across different months), belonging to eight families (Melastomataceae, Piperaceae, Rubiaceae, Monimoaceae, Cyperaceae, Arecaceae, Myrtaceae, and Lauraceae). For our analysis, we used the total of dry fruit weight (biomass) sampled in each grid per month as a measure of the available food resource (Appendix A and Figure A3).

All procedures were authorized for activities with scientific purpose by the Ministry of the Environment—MMA—by the Chico Mendes Institute for Biodiversity Conservation—ICMBio—and by the System of Authorization and Information on Biodiversity—SISBIO: License n: 15733-2.

### 2.8. Data Analysis

To analyze the influence of food availability (arthropods and fruits), climatic variations (temperature and pluviosity), and seasonality (summer and winter) on the number of captures of the species *Euryoryzomys russatus* and *Sooretamys angouya*, Negative Binomial Generalized Linear Mixed Models (GLMMs) were used. This approach was chosen for its robustness in handling overdispersed count data and for accounting for the non-independence of samples through the inclusion of a random effect. Prior to modeling, collinearity among the continuous predictor variables was assessed using a Pearson correlation matrix (r < 0.7), and these variables were standardized (z-score transformed) to ensure comparability of coefficients.

Individual models were constructed for each species, using the number of captures as the response variable. Environmental predictors (arthropod biomass, fruit biomass, temperature, and pluviosity) and season (as a categorical variable: summer vs. winter) were included as fixed effects, while sampling grid (M1, M2 and M3) was included as a random effect to account for spatial dependence. For *E. russatus* and *S. angouya*, the final models retained all predictor variables, including arthropods, fruits, temperature, pluviosity and season (as a categorical variable: summer vs. winter), with the number of captures as the response variable. The GLMMs were fitted using the glmer.nb() function from the lme4 package. Data preparation and manipulation were performed using packages from the tidyverse ecosystem.

Spatially explicit capture-recapture (SECR) models were applied to estimate animal density and analyze spatial patterns of individual movements by incorporating parameters such as detection probability, home range size, and spatial distribution of captures [26]. Each session was initially analyzed as a closed population to estimate species density within individual grids per month. The first 24 capture sessions were also analyzed as an open population to assess long-term trends. The final two sessions (July 2010 and January 2011) were analyzed solely as closed populations due to the extended sampling interval. The null model (M0 NULL) was first applied without interference or data modeling to establish a baseline scenario. However, in sessions with limited data, the model did not fit well, necessitating adjustments. Model selection was guided by Akaike’s Information Criterion (AIC), and the modified Chao model (M0 CHAO MOD) which accounts for heterogeneity in detection probabilities provided the best fit in most sessions with limited data [27]. Other model structures, including heterogeneity models (Mh) and behavioral response models (Mb), were tested but did not outperform M0 CHAO MOD based on AIC values. The program output included estimates of population size (±standard error), variation quotient with 95% confidence intervals, capture probability per session, and variance in meters of capture points. All analyses were conducted in R [28], using version 4.3.1, and the code is available.

## 3. Results

We captured 1838 rodents from 28 species 4782 times. We identified a total of 28 species of non-volant small mammals: 10 species of the order Didelphimorphia, as *Didelphis aurita*, *Gracilinanus microtarsus*, *Marmosops incanus*, *Marmosops paulensis*, *Micoureus paraguayanus*, *Monodelphis americana*, *Monodelphis cunsi*, *Monodelphis iheringi*, *Monodelphis macae*, and *Monodelphis scalops*, and 18 species of the order Rodentia, *Akodon montensis*, *Bibimys labiosus*, *Blarinomys breviceps*, *Brucepattersonius soricinus*, *Delomys sublineatus*, *Euryoryzomys russatus*, *Drymoreomys albimaculatus*, *Juliomys ossitenuis*, *Juliomys pictipes*, *Necromys lasiurus*, *Nectomys squamipes*, *Oligoryzomis nigripes*, *Oligoryzomys flavescens*, *Oligoryzomys* sp., *Oxymycterus judex*, *Sooretamys angouya*, *Thaptomys nigrita*, and *Phyllomys nigrispinus*.

### 3.1. Biological Data

#### 3.1.1. *Sooretamys angouya*

We captured 49 *S. angouya* rodents; 10 of them were recaptured 20 times, totaling 69 captures and recaptures: 13 rodents were captured in M1; 3 rodents in M2; and 33 rodents in M3. Across the three grids, *S. angouya* rodents remained in the grids for an average of 172 ± 89 days; females remained for 180 ± 86 days, whereas males remained for 165 ± 93 days. In total, 31 *S. angouya* females and 18 *S. angouya* males were captured, representing a sex ratio of 1.6:1. The sex ratio for M1, M2, and M3 was 2.2:1, 2:1, and 1.5:1 between females and males, respectively.

We recorded the mean body mass, mean body length, and their respective ranges for females and males of *S. angouya* (Table 1) during the juvenile, subadult, and adult developmental stages. Notably, in this species, females were, on average, consistently larger than males across all three developmental stages (Table 1). Additionally, we calculated the sex ratio for each developmental stage.

**Figure 3:**
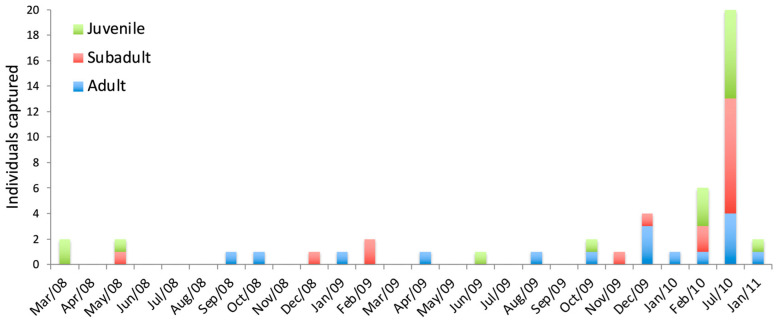
Number of juveniles, subadults, and adults of *Sooretamys angouya* captured during the study at the Reserva Florestal Morro Grande, São Paulo, Brazil.

Juveniles, subadults, and adults were captured throughout most of the year, despite a high capture rate in July 2010 on grid M3. However, for *S. angouya*, there was homogeneity in the number of captures of juveniles (n = 14), subadults (n = 17), and adults (n = 18) throughout the sampling period. Despite the low sample size, all age groups were represented in the population of *S. angouya*, even though capture rates were low (Figure 3).

**Figure A4:**
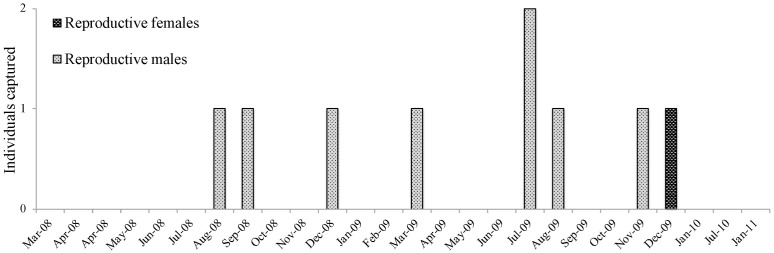
Number of reproductive females and males of *Sooretamys angouya* captured during the study at the Reserva Florestal Morro Grande, São Paulo, Brazil.

Reproductive activity was seldom recorded for this species: only two males were observed with scrotal testes, with one during the summer period (November to March) and the other during the winter period (July to September); one pregnant female was recorded in December 2009 (Appendix A and Figure A4).

#### 3.1.2. *Euryoryzomys russatus*

For *E. russatus*, 207 rodents were captured, with 138 specimens recaptured on 477 occasions, resulting in a total of 684 captures and recaptures. In the grids M1, M2, and M3, 57, 74, and 76 rodents were captured, respectively.

For the three grids grouped, the *E. russatus* remained in the grids for 120 ± 20 days, with the females dwelling for 129 ± 25 days and males for 110 ± 16 days. In the three grids, 83 *E. russatus* females and 124 *E. russatus* males were captured, representing a sex ratio between females and males equal to 1:1.5; the sex ratio found for grids M1, M2, and M3 was: 1:1.8, 1:1.2, and 1:1.5 between females and males, respectively.

We recorded the mean body mass, mean body length, and their respective ranges for females and males of *Euryoryzomys russatus* (Table 1) during the juvenile, subadult, and adult developmental stages. Notably, while females were slightly larger than males in the juvenile and subadult stages, adult males were, on average, larger than adult females (Table 1). Additionally, we calculated the sex ratio for each developmental stage.

**Figure 4:**
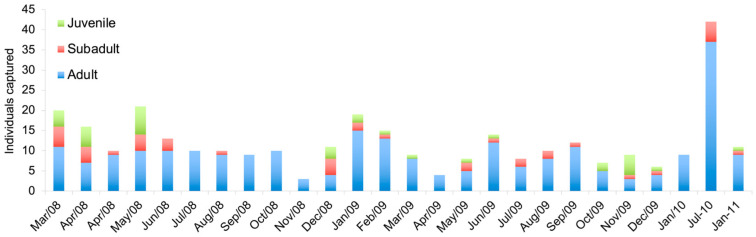
Number of juveniles, subadults, and adults of *Euryoryzomys russatus* captured during the study at the Reserva Florestal Morro Grande, São Paulo, Brazil.

During summer (November to May), there was a populational increase in juvenile and subadult rodents, with adults being captured in all sampling campaigns; in July 2010, there was a peak in the number of adults captured, but in January 2011, the number of rodents returned to levels similar to prior sampling sessions (Figure 4).

**Figure A5:**
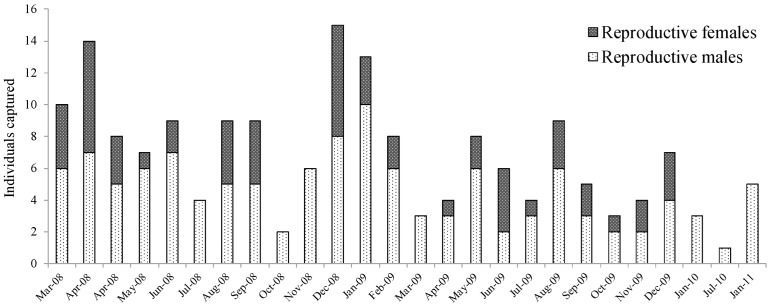
Number of reproductive *Euryoryzomys russatus* females and males captured during the study at the Reserva Florestal Morro Grande, São Paulo, Brazil.

Reproductive males of *E. russatus* were recorded in all sampling months, while females presented reproductive activity in most months sampled (Appendix A, Figure A5).

### 3.2. Capture–Recapture

#### 3.2.1. *Sooretamys angouya*

According to the generalized linear mixed models (GLMMs), none of the abiotic (temperature, precipitation) or biotic factors (arthropod and fruit availability) had a significant effect on the number of captures of *Sooretamys angouya* (*p* > 0.05 for all predictors).

**Figure 5:**
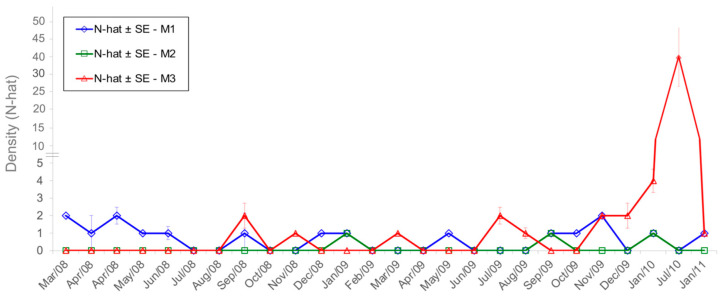
Density (N-hat) ± standard error (SE) for *Sooretamys angouya* in the grids M1, M2, and M3 in the Reserva Florestal Morro Grande, São Paulo, Brazil.

The highest densities for *S. angouya* were in M3. It was not possible to estimate the density for all capture sessions, since we had no captures in some sampling sessions. *S. angouya* had a density of 1.1 ± 1.0 ind./ha (all grids together) in the RFMG, being 0.6 ± 0.5 ind./ha for 14 months in M1, 0.11 ± 0.0 ind./ha for 3 months in M2, and 2.5 ± 2.5 ind./ha for 10 months in M3. The highest density for *S. angouya* was obtained in M3 in July 2010 (31 ± 9.4 ind./ha) during the bamboo blooming of *Merostachys riedeliana* (Poaceae: Bambusoideae) (Figure 5).

Grouping all grids, the average probability of recapture for *S. angouya* in the RFMG was 0.16 ± 0.10%. For *S. angouya*, we had no recaptures in grid M2. The average probability of recapture for *S. angouya* in grid M1 was 0.21 ± 0.13% and 0.28 ± 0.18% for M3. Analyzing the sampling capture stations, the total distance of *S. angouya* capture, on average, was 24.2 ± 9.4 m (range 0–69 m) for all grids grouped. For M1 and M2, the distance was the same—8.2 ± 0 m (ranging from 0 to 8.2 m). For M3, the distance of captures was 30.6 ± 15.5 m (ranging from 0 to 68.8 m).

#### 3.2.2. *Euryoryzomys russatus*

**Figure 6:**
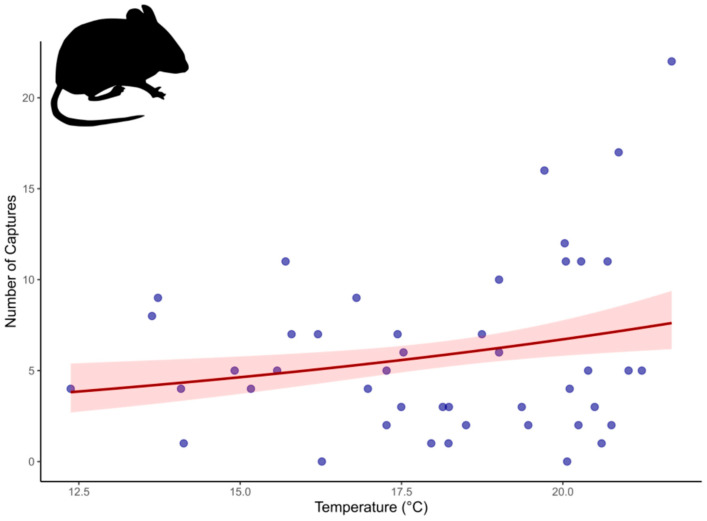
Relationship between temperature and the number of *Euryoryzomys russatus* captures.

According to the Generalized Linear Mixed Models (GLMMs), the number of cap-tures of *Euryoryzomys russatus* was significantly influenced by environmental variables. Specifically, temperature had a positive effect on the number of captures of *E. russatus* (z = 1.997; *p* = 0.0458; R^2^ = 0.045) (Figure 6). On the other hand, no significant associations were observed between the number of captures and arthropod biomass (z = −1.696; *p* = 0.0898) or fruit biomass (z = 1.061; *p* = 0.2889).

**Figure 7:**
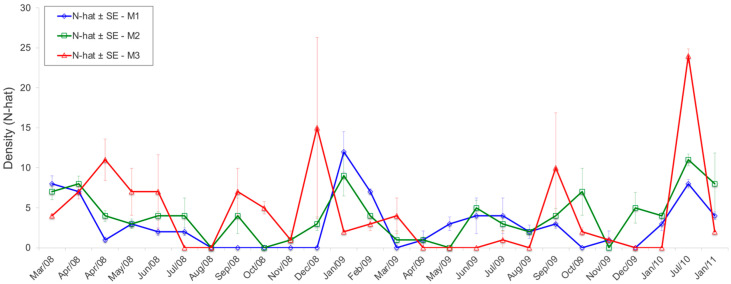
Density (N-hat) ± standard error (SE) for *Euryoryzomys russatus* in the grids M1, M2, and M3 in the Reserva Florestal Morro Grande, São Paulo, Brazil.

*E. russatus* had a density of 4.3 ± 3.7 ind./ha (for all grids pulled together) in the RFMG, being 3.4 ± 2.7 for 18 months in M1, 4.3 ± 2.5 for 22 months in M2, and 5.1 ± 5.0 ind./ha for 18 months in M3. The highest density was recorded in July 2010 in grid M3 (24 ± 0.7 ind./ha) and is related to the flowering and fruiting of the bamboo species *Merostachys riedeliana* (Poaceae: Bambusoideae) (Figure 7).

The average probability of recapture for *E. russatus* grouping all grids of the RFMG was 0.33 ± 0.19% and separately was 0.33 ± 0.18% for M1, 0.35 ± 0.18% for M2, and 0.3 ± 0.2% for M3. The average capture distance for *E. russatus* in all grids was 42.2 ± 29.4 m, ranging from 0 to 125 m. The capture distance was 41.8 ± 30.6 m, 44.2 ± 31.7 m, and 40.2 ± 25.5 m for M1, M2, and M3, respectively.

## 4. Discussion

Our results partially support the proposed hypotheses. As predicted, *Euryoryzomys russatus* and *Sooretamys angouya* exhibited distinct responses to environmental variation, reflecting their contrasting ecological strategies. *E. russatus* showed a significant association with temperature, suggesting sensitivity to seasonal climatic variation, consistent with its classification as a more forest-dependent species. In contrast, *S. angouya* did not show strong associations with any of the tested environmental variables, supporting the hypothesis that it possesses greater ecological plasticity. *Euryoryzomys russatus* was more abundant and exhibited greater mobility than *Sooretamys angouya* at RFMG. While *E. russatus* had a male-biased sex ratio, *S. angouya* showed a female-biased ratio. Both species persisted in the grids for extended periods, but only *E. russatus* displayed clear seasonal fluctuations, with juvenile and subadult numbers peaking in summer. Reproductive *E. russatus* males were present year-round, whereas *S. angouya* exhibited reproductive activity in only two distinct seasons. Population densities of both species peaked in July 2010, coinciding with the bamboo flowering event (see [29]). Recapture probabilities were higher for *E. russatus* than *S. angouya*, reflecting its greater movement and possibly higher detectability. Overall, *E. russatus* demonstrated more pronounced seasonal population dynamics and environmental responsiveness, while *S. angouya* maintained a more stable but lower-density population. Our findings on population parameters such as density, recapture rates, and movement patterns varied across the grids, with distinct trends evident in M1 (reforested), M2 (selectively logged), and M3 (structured forest), underscoring the influence of specific habitat characteristics on these sympatric rodent species.

### 4.1. Capture Efficiency and Trap Effectiveness

Our study captured *E. russatus* and *S. angouya* rodents at different life stages using Sherman and pitfall traps. Sherman traps were responsible for over 75% of *E. rusatus* and 50% of *S. angouya* captures, predominantly adults, whereas pitfall traps captured the remaining rodents, including juveniles and subadults. The higher capture frequency of juveniles may be partly due to their lower escape ability, as adult rodents might be more capable of escaping the trap structure. These findings align with previous studies on population dynamics, confirming that Sherman traps are more effective for adults while pitfall traps are crucial for younger rodents [16,30,31,32].

### 4.2. Population Structure and Dynamics

Our results indicate distinct population structures between these sympatric rodents. *Euryoryzomys russatus* was more abundant than *S. angouya*, which exhibited low densities (0–2 ind./ha). Seasonal variations were evident: *S. angouya* was more frequently captured in summer, whereas *E. russatus* peaked in winter. Reproductive *E. russatus* males were present year-round, while reproductive females were more common in summer. In contrast, only one pregnant *S. angouya* female was recorded. Juveniles and subadults of both species were primarily captured in summer. The sex ratio was skewed towards females in *S. angouya* and males in *E. russatus*. *S. angouya* individuals showed higher grid permanence and moved longer distances between captures than *E. russatus*.

### 4.3. Capture Success and Habitat Influence

The capture success of *Euryoryzomys russatus* at RFMG (0.87%), although lower than that in some well-preserved forests [33,34,35], exceeded the rates from forest fragments [16,32]. This finding is consistent with the ecology of *E. russatus*, a species more abundant in continuous forests or large, connected forest fragments and with low occurrence in intensely fragmented landscapes [13,36,37], where its capture success reflects habitat quality [6]. In contrast, the low capture success of *Sooretamys angouya* (0.08%), similar to other studies in the Atlantic Forest [33,38,39,40], can be interpreted in light of its distinct response to fragmentation. *S. angouya* tends to be less abundant than *E. russatus* in continuous forests but can exhibit higher relative abundance in fragmented environments [14,16]. Thus, the higher detectability of *E. russatus* compared to *S. angouya* at RFMG suggests that the study area possesses good habitat integrity, favoring species more associated with continuous forests, in line with their different ecological strategies in response to fragmentation.

### 4.4. Grid Permanence and Sex Ratios

*S. angouya* females remained in the grids longer than males, while *E. russatus* females outlasted males. In Santa Catarina, rodents of both species exhibited shorter grid residency [33]. While no significant difference in grid permanence between sexes was observed, females remained slightly longer, consistent with previous studies [32,41,42]. This pattern may be linked to dispersal capacity, food availability, and reproduction [34,43].

Sex ratio patterns varied by species and age in RFMG. *S. angouya* had a female-biased ratio, opposite to findings in Santa Catarina [33]. *Euryoryzomys russatus* exhibited a male-biased ratio, consistent with prior research [33,35,44]. Juvenile *S. angouya* had a 6:1 female-to-male ratio, while subadults (1.1:1) and adults (1.2:1) showed a more balanced distribution. *Euryoryzomys russatus* juveniles had a near-equal ratio (1:1.1), shifting to a male bias in subadults (1.9:1) and adults (1:2.1). These fluctuations suggest higher male dispersal or mortality at younger ages, with adult sex ratios balancing due to habitat learning and reduced predation risk [45,46,47]. Higher female mortality in adults could be linked to pregnancy-related vulnerability [46]. Male-biased captures in adults may reflect increased movement during the reproductive season [43,48]. This pattern may be linked to differential survival rates, reproductive investment, or demographic compensation within the population, though further studies would be needed to confirm such mechanisms.

### 4.5. Body Size, Reproduction, and Resource Availability

Adult *S. angouya* and *E. russatus* females were equal to or slightly larger than males, a pattern similar to that observed in *Nephelomys albigularis* a Neotropical species also with a more generalist and omnivorous diet, whose sexes exhibit similar head and body lengths (102–162 mm) [49]. In contrast, *E. russatus* exhibited a size dimorphism where adult males were, on average, larger than adult females. This pattern, detailed in our results, may result from sex-specific energy allocation to growth and reproduction [50,51]. *E. russatus* females were reproductively active year-round, except in certain months, while reproductive males were captured throughout the study period. Seasonal reproductive peaks in summer were likely tied to increased fruit availability [51,52]. Our findings support previous research indicating continuous reproduction with peaks in resource-rich periods [48,51,53,54].

For *Euryoryzomys russatus*, temperature had a significant effect on capture rates, with the number of captures increasing as temperatures rose. This pattern may suggest that the species exhibits higher metabolic and foraging activity during warmer periods. This could be linked to increased resource availability or more favorable conditions for reproduction and dispersal. Conversely, no effect was observed for precipitation, which is consistent with previous findings from Santa Catarina [33]. The lack of association with arthropod biomass may reflect a predominantly plant-based diet, as it’s often described in the literature as a frugivorous/granivorous animal [48,51,55,56]. In contrast, *Sooretamys angouya* capture rates showed no significant association with either biotic or abiotic variables, aligning with prior studies [33]. This apparent ecological stability might be related to its generalist feeding behavior and broader tolerance to environmental variation [57], or to unmeasured factors such as predation pressure or interspecific interactions.

Overall, our results support the hypothesis that the two species respond differently to environmental drivers, with *E. russatus* showing stronger sensitivity to climatic and resource variation, while *S. angouya* appears more ecologically plastic. According to the São Paulo State Red List (Decree No. 63.853, 27 November 2018), *Euryoryzomys russatus* is currently classified as Near Threatened (NT), while *Sooretamys angouya* is listed as Least Concern (LC) [5]. These classifications are supported by our findings and reinforce the importance of considering species-specific ecological responses when developing conservation strategies.

## 5. Conclusions

Our findings reveal that *Euryoryzomys russatus* and *Sooretamys angouya*, although sympatric, display distinct ecological strategies shaped by seasonal reproductive patterns, sex-specific behavior, and differing responses to environmental factors. For *E. russatus*, capture rates were influenced by temperature, suggesting a specific sensitivity to climatic conditions. These species-specific traits suggest niche differentiation that potentially reduces direct competition and facilitates coexistence. Moreover, their contrasting responses to habitat quality and fragmentation highlight their differential roles within the small mammal community— *S. angouya* possibly acting as a more generalist species and *E. russatus* as a more habitat-sensitive indicator. Recognizing such ecological roles is crucial to understanding community structure and resilience in fragmented landscapes. Future research should therefore focus not only on further exploring their demographic and spatial dynamics but also on their ecological interactions within the broader community. This knowledge is fundamental for designing effective, tailored conservation and management strategies under ongoing anthropogenic pressures.

## Figures and Tables

**Table 1:** Average body mass (g), body length (mm), sample size (n), and sex ratio (F:M) of *Sooretamys angouya* and *Euryoryzomys russatus* rodents captured in the RFMG, classified by developmental stage (juvenile, subadult, and adult) and sex. Ranges are provided in parentheses.

Species	Stage	Sex	Avg. Body Mass (g) (Range)	n	Avg. Body Length (mm) (Range)	Sex Ratio (F:M)
***S. angouya***	Juvenile	Female	27 (15–36)	12	98 (80–133)	6:1
		Male	23 (22–24)	2	97.5 (90–105)	
	Subadult	Female	54.2 (33.5–66)	9	122 (112–130)	1.1:1
		Male	51 (36–60)	8	120 (106–130)	
	Adult	Female	99.1 (56.1–158)	10	148 (120–169)	1.2:1
		Male	85.1 (70.1–116)	8	146 (132–157)	
***E. russatus***	Juvenile	Female	18.9 (10–31)	15	89 (76–101)	1:1.1
		Male	17.2 (12.5–28)	17	83 (71–95)	
	Subadult	Female	38.9 (19–63)	23	110 (85–130)	1.9:1
		Male	35.6 (26–56)	12	108 (89–127)	
	Adult	Female	62.7 (42–104)	45	132 (94–150)	1.2:1
		Male	69.8 (38–128)	95	133 (89–161)	

**Figure A1:**
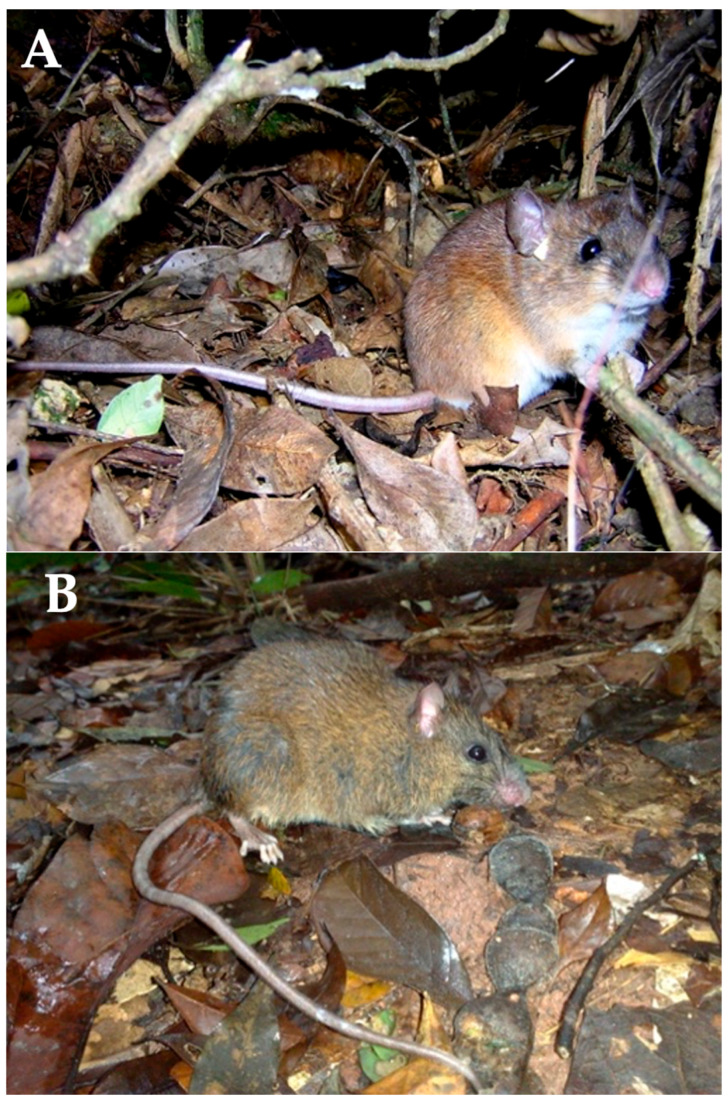
Oryzomyini rodent species present in the São Paulo Atlantic Forest Plateau; (**A**) *Euryoryzomys russatus*, (**B**) *Sooretamys angouya*.

## Data Availability

Publicly available datasets were analyzed in this study. This data can be found here: https://doi.org/10.5061/dryad.pg4f4qs04.
